# Automated liver and spleen segmentation for MR elastography maps using U-Nets

**DOI:** 10.1038/s41598-025-95157-w

**Published:** 2025-03-28

**Authors:** Noah Jaitner, Jakob Ludwig, Tom Meyer, Oliver Boehm, Matthias Anders, Biru Huang, Jakob Jordan, Tobias Schaeffter, Ingolf Sack, Rolf Reiter

**Affiliations:** 1 Department of Radiology, Charité-Universitätsmedizin Berlin, corporate member of Freie Universität Berlin and Humboldt-Universität zu Berlin, Hindenburgdamm 30, 12203 Berlin, Germany; 2 Division of Medical Physics and Metrological Information Technology, Physikalisch- Technische Bundesanstalt, Abbestr. 2-12, 10587 Berlin, Germany; 3 Department of Medical Engineering, Technical University Berlin, Straße des 17. Juni 135, 10623 Berlin, Germany; 4 Berlin Institute of Health at Charité-Universitätsmedizin Berlin, BIH Biomedical Innovation Academy, BIH Charité Digital Clinician Scientist Program, Charitéplatz 1, 10117 Berlin, Germany

**Keywords:** Biomarkers, Gastroenterology, Medical research, Medical imaging, Magnetic resonance imaging

## Abstract

To compare pretrained and trained U-Nets for liver and spleen segmentation in multifrequency magnetic resonance elastography (MRE) magnitude images for automated quantification of shear wave speed (SWS). Seventy-two healthy participants (34 ± 11 years; BMI, 23 ± 2 kg/m^2^; 51 men) underwent multifrequency MRE at 1.5T or 3T. Volumes of interest (VOIs) of liver and spleen were generated from MRE magnitude images with mixed T2-T2* image contrast and then transferred to SWS maps. Pretrained and trained 2D and 3D U-Nets were compared with ground truth values obtained by manual segmentation using correlation analysis, intraclass correlation coefficients (ICCs), and Dice scores. For both VOI and SWS values, pairwise comparison revealed no statistically significant difference between ground truth and pretrained and trained U-Nets (all *p* ≥ 0.95). There was a strong positive correlation for SWS between ground truth and U-Nets with *R* = 0.99 for liver and *R* = 0.81–0.84 for spleen. ICC was 0.99 for liver and 0.90–0.92 for spleen, indicating excellent agreement for liver and good agreement for spleen for all U-Nets investigated. Dice scores showed excellent segmentation performance for all networks with the 2D U-Net achieving slightly higher values for the liver (0.95) and spleen (0.90), though the differences between the three tested U-Nets were minimal. The excellent performance we found for automated liver and spleen segmentation when applying 2D and 3D U-Nets to MRE magnitude images suggests that fully automated quantification of MRE parameters within anatomical regions is feasible by leveraging the previously unexploited anatomical information conveyed in MRE magnitude images.

## Introduction

Magnetic resonance elastography (MRE) is used clinically as a noninvasive method for grading hepatic fibrosis and is extensively studied for use in most organs of the human body including the liver and spleen^1–4^. MRE characterizes the biophysical properties of biological tissues with stiffness being the most commonly investigated quantitative parameter. A variety of studies have shown that hepatic stiffness increases with the stage of fibrosis and that spleen stiffness can also be affected by progressive liver disease and portal hypertension^5–11^. Therefore, an assessment of both liver and spleen is warranted in many research settings. Accurate manual segmentation of 3D liver and spleen datasets, preferably performed by a board-certified radiologist, is time-consuming and costly.

U-Nets, which are a special type of deep convolutional neural networks (CNNs), have been applied to a variety of anatomical segmentation tasks due to their ability to generalize image-based processing tasks^12^. In contrast to non-machine learning-based approaches they are capable of providing accurate segmentation across different organs without the necessity of initial assumptions or manual tuning^13,14^. For the liver and spleen, automated segmentation is often performed on conventional T1- or T2-weighted images with subsequent transfer to other MRI sequences from the same dataset or even computed tomography using trained U-Net CNNs and transfer learning^15–17^. Unlike such conventional MR images, MRE maps are reconstructed from phase images and averaged over a period of around 5 min in free breathing or multiple short 20-s breath-holds^5,6,18,19^. MRE magnitude images have been largely unexploited, even though they provide conventional morphological anatomy in images with mixed T2/T2* contrast acquired using spin-echo sequences with fat saturation. Despite an abundance of CNN-based methods for automated segmentation, there is a lack of dedicated algorithms for the segmentation of MRE maps^20^. Fully exploiting the anatomical and viscoelastic information from a single MRE dataset could facilitate data analysis without co-registration artifacts and allow automated postprocessing of MRE parameters.

Pretrained models may increase segmentation performance when data size is limited^21^. Therefore, our aim was to compare pretrained and trained U-Nets for liver and spleen segmentation of MRE magnitude images.

## Methods

### Participants

The study was approved by the Institutional Review Board of Charité – Universitätsmedizin Berlin and was conducted in accordance with relevant guidelines and regulations after obtaining written informed consent from participants. This study presents a secondary assessment of prospectively acquired data^22^ and additionally acquired data. A total of 72 healthy participants were included (21 women, 51 men). Mean age was 34 ± 11 years, and mean body mass index (BMI) was 23 ± 2 kg/m^2^. Demographic data are summarized in Table 1.

### MRE protocols

Images were acquired on two different MRI scanners. Fifty participants were investigated in a 3T MRI scanner (Magnetom Lumina, Siemens Healthineers, Germany) with a 12-channel receiver coil. 3D MRE was performed using a Cartesian, single-shot, spin-echo, echo-planar imaging MRE sequence^23^ at three frequencies (25 Hz, 31.25 Hz, 40 Hz). Images were acquired during three separate 20-s breath-holds in 37 participants and during free breathing in the remaining 13 participants. Images acquired during 20-s breath-holds had a voxel size of 3 × 3 × 3 mm^3^ versus 2.5 × 2.5 × 3 mm^3^ for images acquired during free breathing. Further imaging parameters were as follows: field of view, 360 × 258 mm^2^; 11 slices, motion-encoding frequency, 61.43 Hz; amplitude of the motion-encoding gradient, 34 mT/m; repetition time, 910 ms; echo time, 45 ms. Twenty-two participants underwent imaging on a 1.5T MRI scanner (Magnetom Aera, Siemens Healthineers, Germany) with an 18-channel receiver coil. The same 3D MRE sequence as described above was used with four frequencies (30 Hz, 40 Hz, 50 Hz, 60 Hz), and images were acquired during 5 min of free breathing. Further imaging parameters were as follows: voxel size, 3 × 3 × 5 mm^3^; field of view, 380 × 308 mm^2^; 19 slices, motion-encoding frequency, 33.29–66.67 Hz; amplitude of the motion-encoding gradient, 20 mT/m; repetition time, 2730 ms; echo time, 76 ms. Quantitative maps of shear wave speed (SWS in m/s) were generated using the tomoelastography processing pipeline publicly available at https://bioqic-apps.charite.de^24–26^. The ground truth of liver and spleen segmentation in multifrequency MRE data was established by a board-certified radiologist (R.R.) with over 10 years of experience in abdominal MRE, who performed manual segmentation to generate volumes of interest (VOIs) using ITK-SNAP (3.6.0, University of Pennsylvania, USA).

**Table 1:** Summary of participant data.

Characteristic	3T MRI	1.5T MRI
No. of participants	50	22
Age (y)	32 ± 8	40 ± 12
Women/men	10/40	11/11
BMI (kg/m^2^)	23 ± 2	23 ± 3
Frequencies (Hz)	25, 31.25, 40	30, 40, 50, 60

*BMI*, body mass index.

### U-Net

**Fig. 1:**
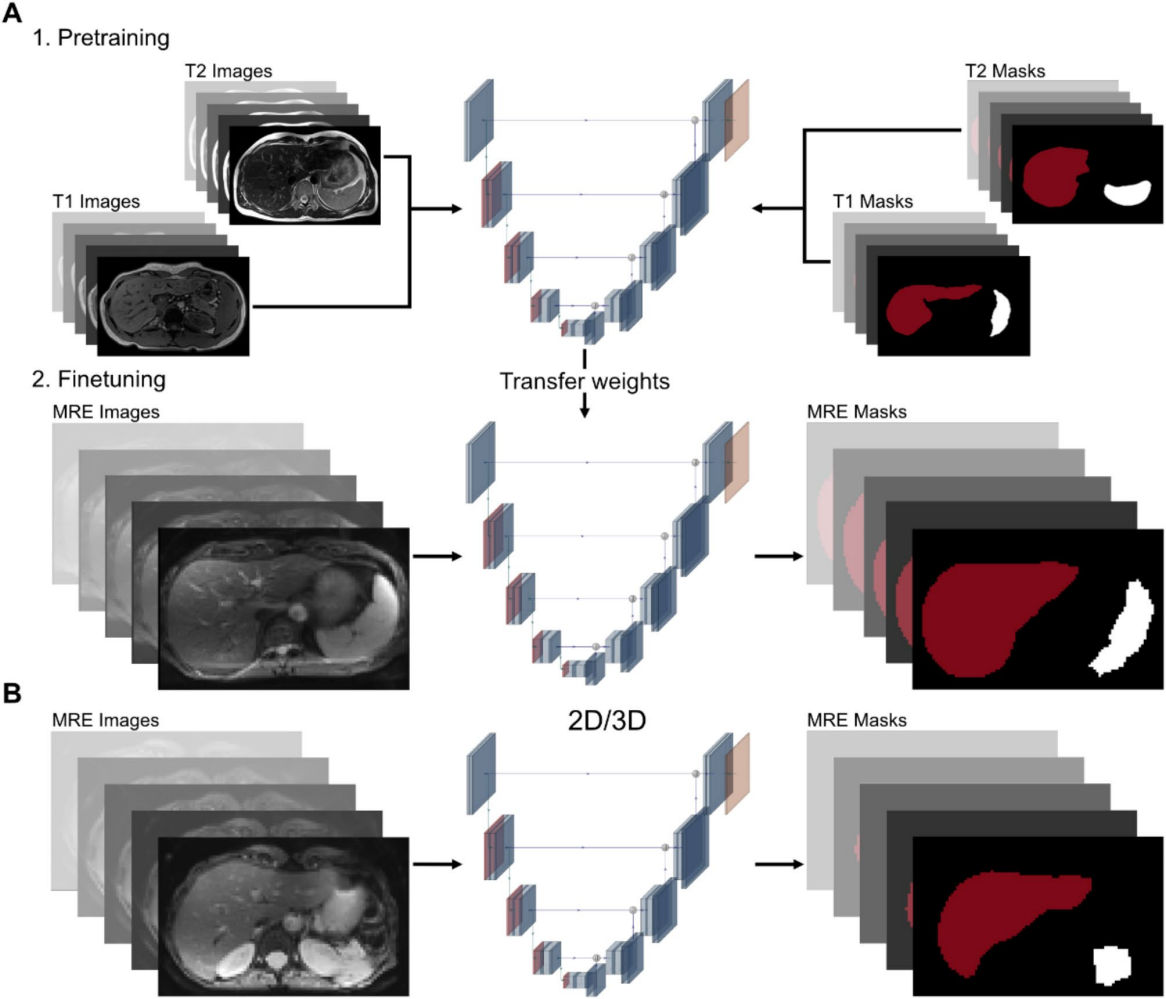
Overview of training procedure. **(a)** For the pretrained model, the U-Net was first trained on T1- and T2-weighted images; the weights were then transferred and additional finetuning was performed with MRE data. **(b)** For the 2D and 3D U-Net, the network was directly trained on the MRE images.

The training procedure is shown in Fig. 1. Training was performed with the nnU-Net framework^27^ on a dedicated graphics processing unit (NVIDIA A100 80G, NVLink-connected). The data were randomly selected and divided into 46 training cases (64%), 11 validation cases (16%), and 15 test cases (20%). Data augmentation was performed by creating and rotating patches to expand the small training dataset. Training was conducted using a 5-fold cross-validation. The pretrained network was publicly available and was previously trained on T1- and T2-weighted MRI data^27^. The weights were transferred and additionally trained on MRE data. The 2D and 3D U-Nets were trained on MRE data only. Optimization was performed using the Adam optimizer with an initial learning rate of 0.01^28^. Training was stopped after 1000 epochs, and LeakyRelu was chosen as an activation function for all three network types^29^. In the first encoding step, 32 hidden layers were chosen for all three network types. The pretrained network had 7 encoding steps and 320 hidden layers in the final step. The 3D U-Net had 5 encoding steps with 320 hidden layers in the final step. The 2D U-Net had 5 encoding steps with 512 hidden layers in the last step. The pretrained model took 67 h to train, 3 min to validate, and 9.2 s to test. The 2D U-Net took 26 h to train, 2 min to validate, and 3 s to test. The 3D U-Net took 5 h to train, 1 min 40 s to validate, and 2 s to test.

Both manual segmentation of VOIs to establish the ground truth and U-Net training were performed on MRE magnitude images^20,30^. The VOIs derived from the magnitude images were then transferred to the SWS maps and voxels whose value were lower than a threshold of 1 m/s corresponding to larger vessels were excluded^31^. Dice scores and Hausdorff distances were calculated to evaluate segmentation accuracy.

Uncertainty of test data inferred by a trained model was estimated using deep ensembles uncertainty quantification^32^. This method provides the most accurate uncertainty results for quantification techniques. The outcome for deep ensembles estimates epistemic (model) uncertainty. Using Bayesian probability theory^33^, we can track epistemic and aleatoric uncertainty from the probability distribution

1 $$p({\varvec{y}}|{\varvec{x}},~D)~=~\smallint \underbrace {{p({\varvec{y}}|{\varvec{x}},~\theta )}}_{{{\text{Aleatoric}}}}~\underbrace {{p(\theta |D)}}_{{{\text{Epistemic}}}}d\theta ,$$

where ${\varvec{x}}$ and ${\varvec{y}}$ represent input data and prediction labels, respectively, $\theta$ the U-Net model parameters, and *D* the training dataset. The equation shows that we can influence epistemic but not aleatoric uncertainty by changing $\theta$ during the training process. For computing the final uncertainty score per prediction, we estimated the average over $p({\varvec{y}}|{\varvec{x}},~D)$ for the liver, spleen, and negative background classes. Finally, we calculated the variance over all ensemble members with the standard deviation as a metric.

### Statistical analysis

Mean and standard deviation were used to report continuous variables and group values. Group values (ground truth, pretrained, 2D U-Net, 3D U-Net) were tested for differences using the Kruskal-Wallis test followed by a pairwise Wilcoxon ranksum test as post-hoc analysis. Subgroup analysis was performed using the Wilcoxon-Mann-Whitney-Test. VOI and SWS estimates were compared between manual and automated segmentation by Pearson correlation and Bland-Altman analysis. Intraclass correlation coefficients (ICCs) with 95% confidence interval (CI) were established. ICCs were categorized as: < 0.5 indicating poor agreement, 0.5–0.75 indicating moderate agreement, 0.75–0.9 indicating good agreement, and 0.9-1.0 indicating excellent agreement^34^. The significance level was set at 5%. Since this is a pilot study, no reliable prior information on possible effect sizes was available, and we justify the sample size for pragmatic reasons and do not conduct post-hoc power calculations^35^. Statistical analysis was performed in R (Version 4.0.3, R-Foundation, Vienna, Austria).

## Results

**Fig. 2:**
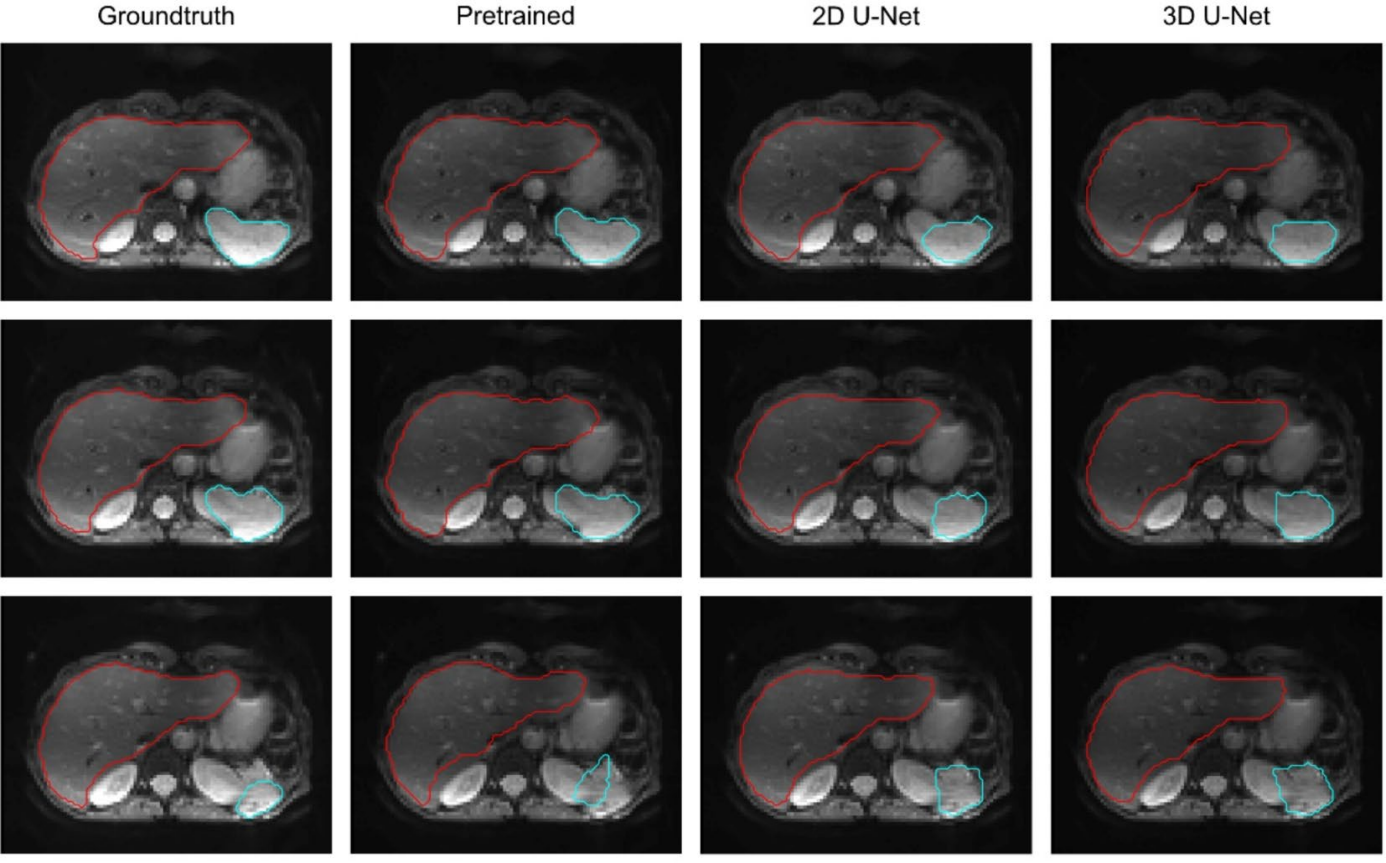
Segmentation of MRE magnitude images from three slices acquired in a 61-year-old female healthy participant. Segmentation results for the liver are shown in red and for the spleen in turquoise.

**Fig. 3:**
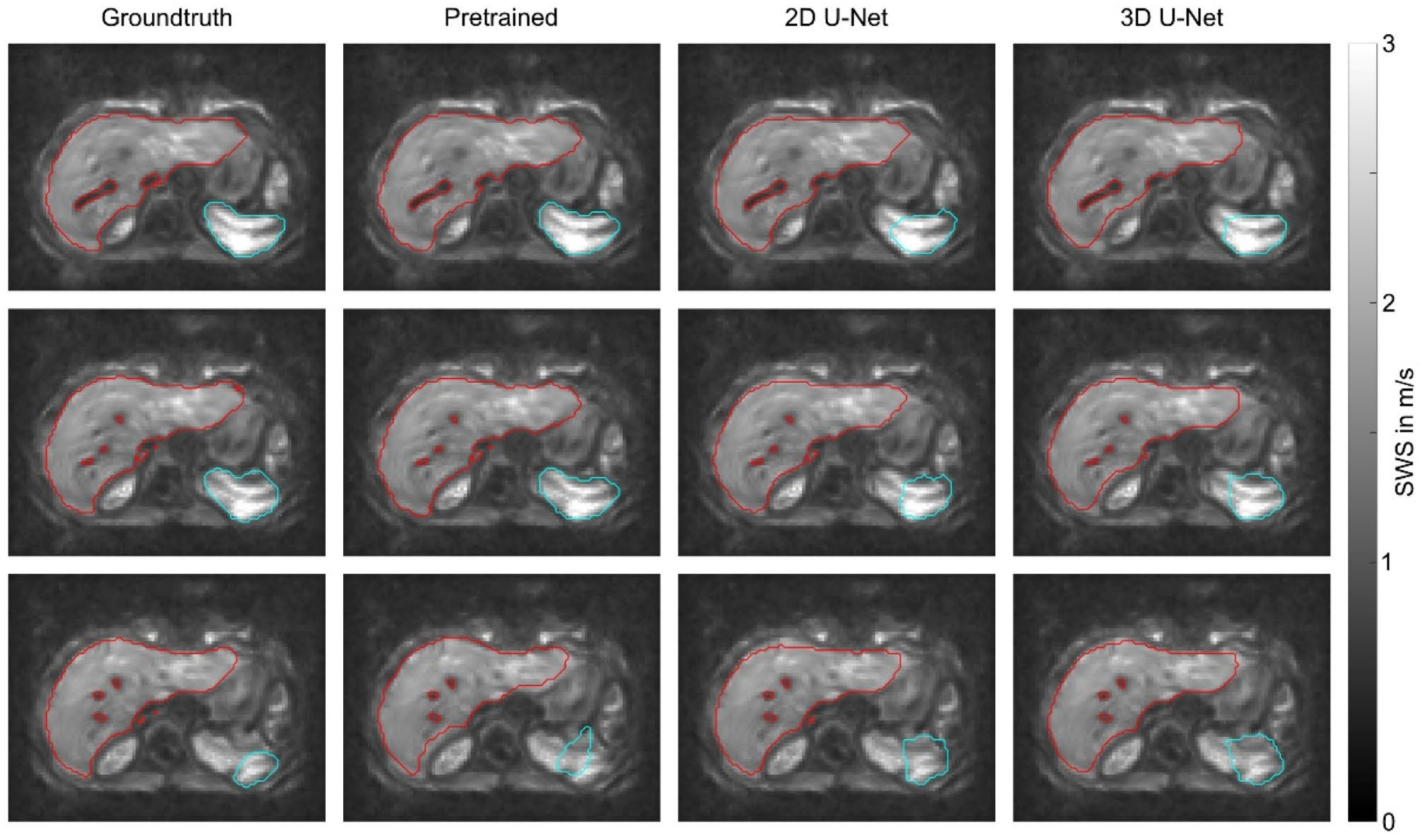
Segmentation of shear wave speed (SWS) maps of three slices of the same participant as shown in Fig. 2. Segmentation results for the liver are shown in red and for the spleen in turquoise. A threshold of 1 m/s was used to remove vessels.

Figure 2 presents examples of liver and spleen segmentation on MRE magnitude images. Following segmentation, the VOIs defining the ground truth and those provided by the pretrained and trained U-Nets were transferred to the corresponding SWS maps, as shown in Fig. 3.

### Liver

**Fig. 4:**
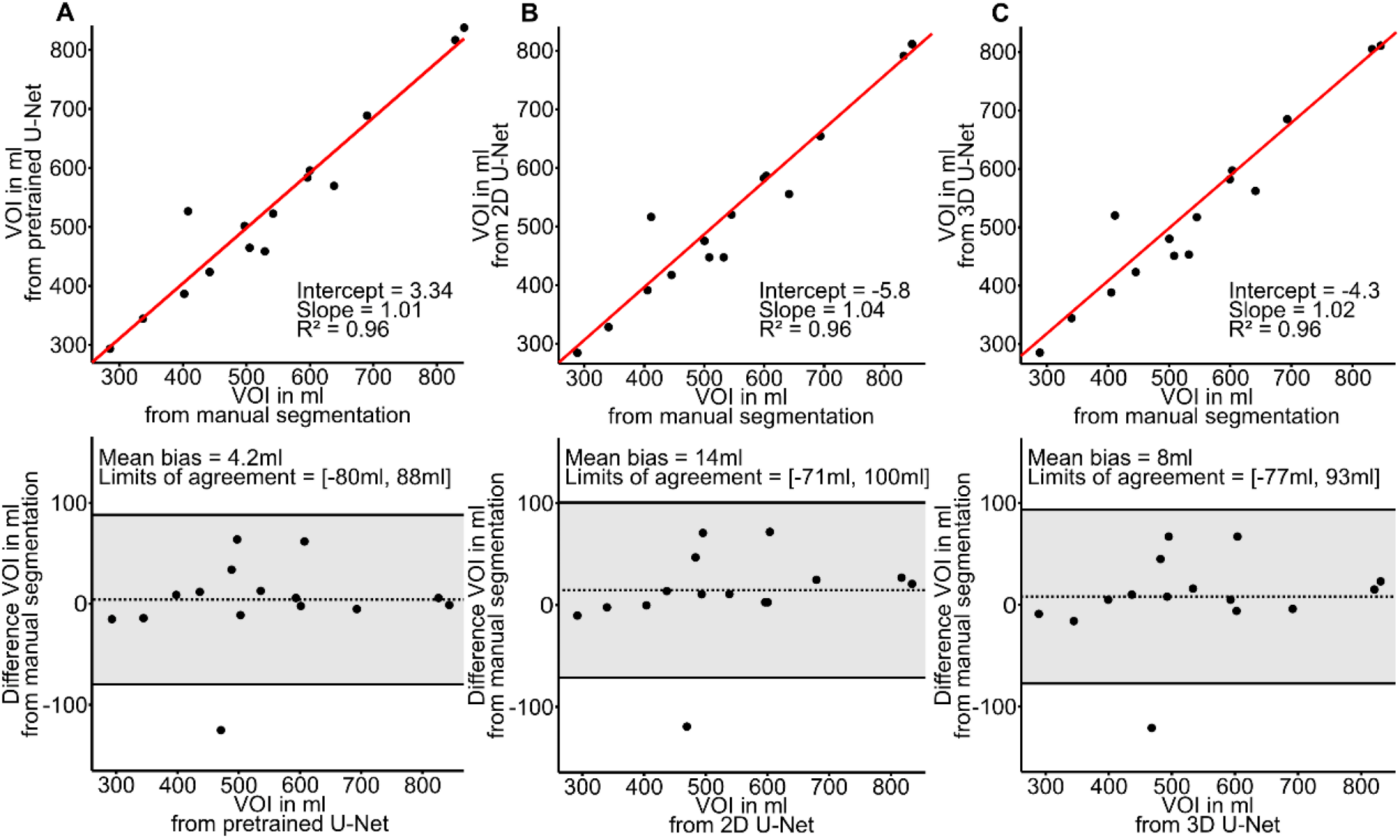
Liver. Agreement between three different U-Nets and ground truth for VOI. Linear regression and Bland-Altman analysis for segmentation computed by pretrained network **(a)**, by 2D U-Net **(b)**, and by 3D U-Net **(c)**.

**Fig. 5:**
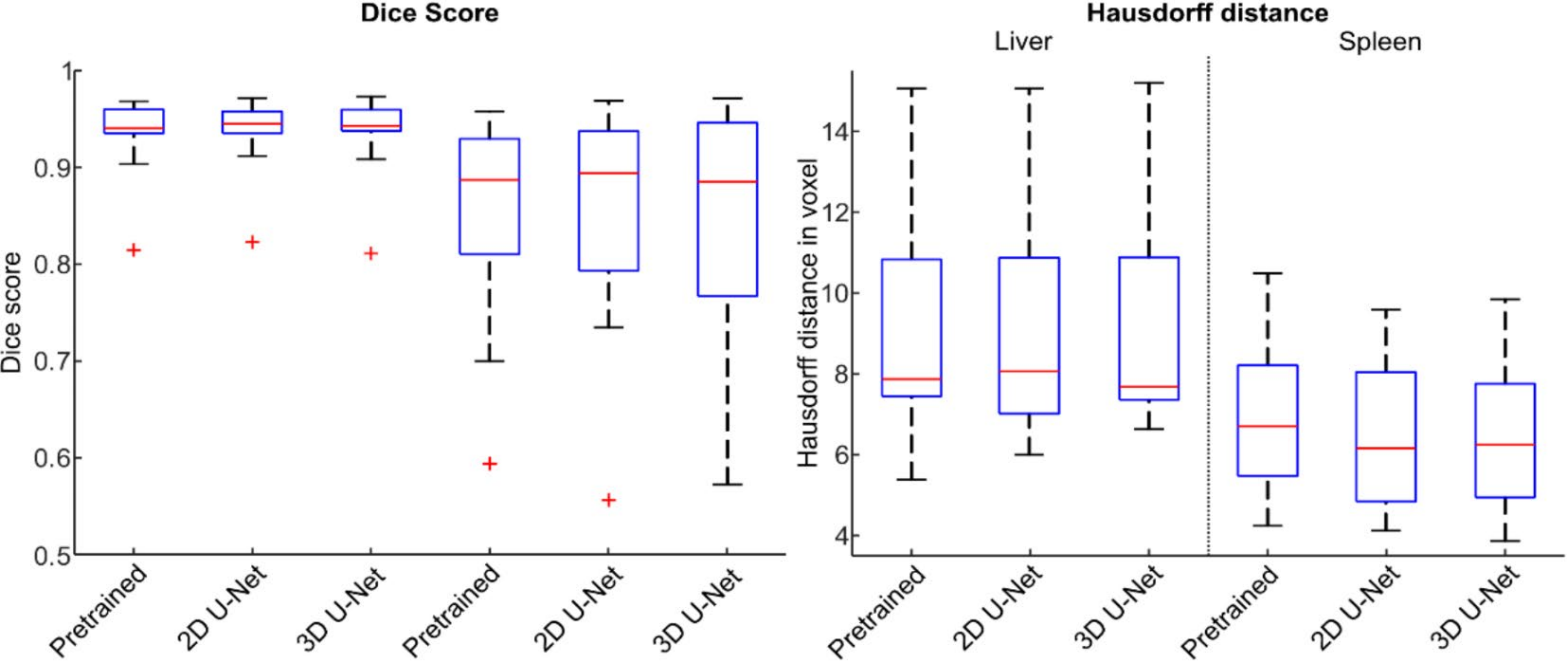
Dice scores and Hausdorff distances for liver and spleen segmentation by the three network architectures applied to unseen test data.

**Fig. 6:**
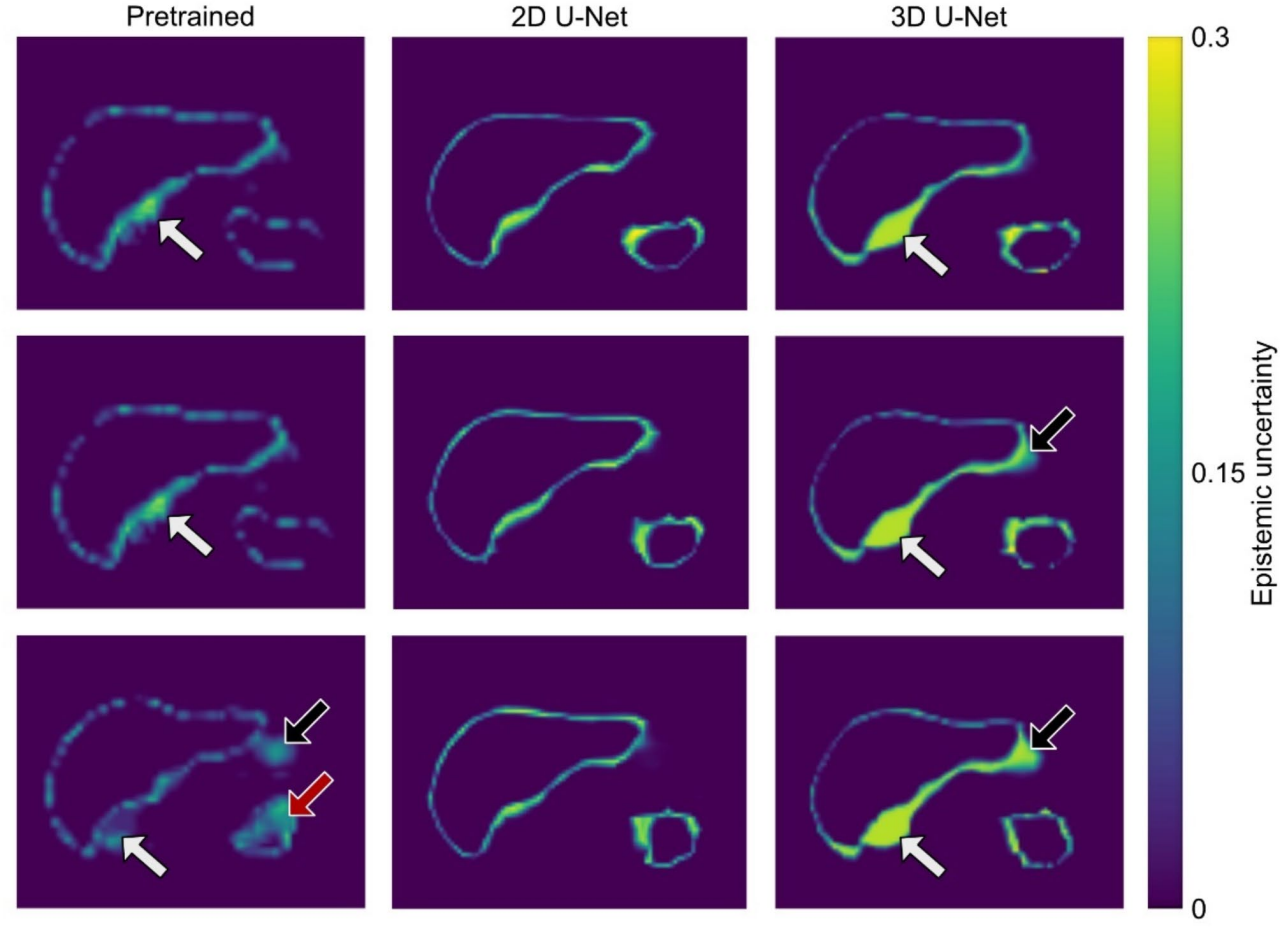
Epistemic uncertainty of network ensemble according to Eq. (1) of the same participant as shown in Figs. 2 and 3. Larger values indicate a higher standard deviation of softmax activation probabilities between ensemble members. Pronounced uncertainty appears at the adjacent right kidney (white arrow) and at the stomach (black arrow) for pretrained and 3D U-Net. The pretrained model shows homogenous uncertainty for the spleen in the last slice (red arrow).

Mean liver VOI size was 541 ± 157 ml, 537 ± 151 ml, 526 ± 146 ml, and 533 ± 148 ml for ground truth, pretrained, 2D U-Net, and 3D U-Net, respectively, without a statistically significant difference (*p* = 0.99). Figure 4 shows VOI values from manual segmentation compared with those obtained from U-Nets. As shown in Fig. 5, the mean liver Dice score was 0.94, 0.95, 0.94, while mean liver Hausdorff distance was 7.87 voxels, 8.06 voxels, and 7.68 voxels for pretrained, 2D U-Net, and 3D U-Net, respectively. Subgroup analysis based on field strength, breathing pattern, and frequency revealed no statistically significant differences in Dice scores. Mean liver SWS was 1.35 ± 0.13 m/s, 1.35 ± 0.12 m/s, 1.35 ± 0.12 m/s, and 1.35 ± 0.13 m/s for ground truth, pretrained, 2D U-Net, and 3D U-Net, respectively, without a statistically significant difference (*p* = 0.99). Pairwise comparisons of *p*-values of post-hoc analysis are listed in Table 2. In the epistemic uncertainty maps in Fig. 6, we observed typical uncertainty features such as the liver segment boundaries. Furthermore, an out-of-distribution area with a high uncertainty is highlighted at the adjacent right kidney. In some cases, the stomach shows similar homogeneous intensity, and the model is uncertain about the transition from the liver. This inaccuracy also leads to pronounced uncertainty in the upper right portion of the organ.

**Fig. 7:**
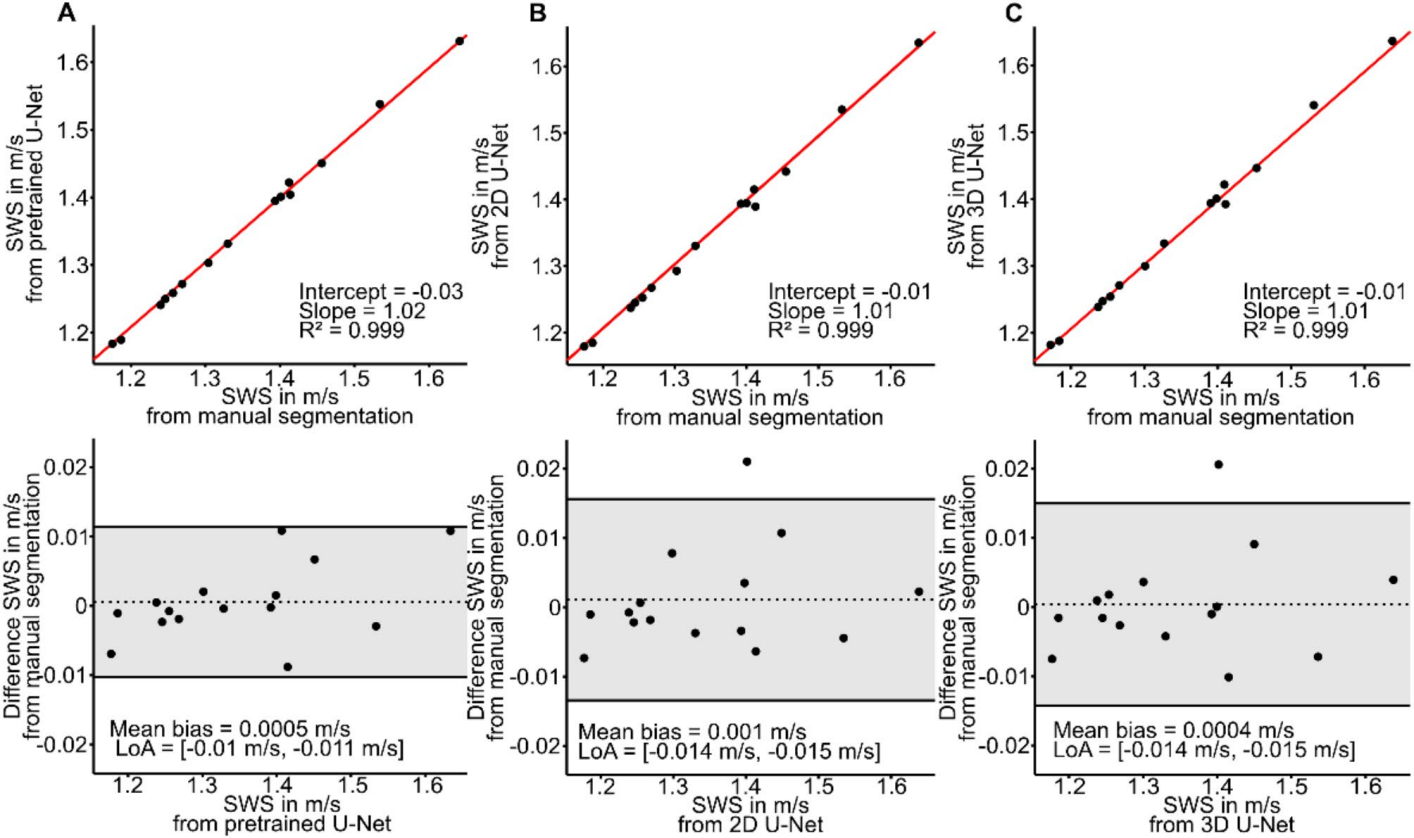
Liver. Agreement between three different U-Nets and ground truth for SWS. Linear regression and Bland-Altman analysis for segmentation computed by pretrained network **(a)**, by 2D U-Net **(b)**, and by 3D U-Net **(c)**.

Figure 7 shows averaged SWS values obtained from manual segmentations compared with those obtained from U-Nets. Mean SWS values from manual segmentation correlated closely with the mean values from the pretrained network (*R* = 0.99, slope = 1.02, intercept = − 0.03), 2D U-Net (*R* = 0.99, slope = 1.01, intercept = − 0.01), and 3D U-Net (*R* = 0.99, slope = 1.01, intercept = − 0.01). There was a small bias of 0.0005 m/s, 0.001 m/s, and 0.0004 m/s for pretrained, 2D U-Net, and 3D U-Net, respectively. 95% limits of agreement (LoA) were (− 0.01) to 0.011 m/s, (− 0.014) to 0.015 m/s, and (− 0.014) to 0.015 m/s for pretrained, 2D U-Net, and 3D U-Net, respectively. The ICC was 0.99 for all three network types, showing excellent agreement.

**Table 2:** Summary of parameters for and post-hoc *p*-values (in parentheses, pairwise comparison with ground truth) obtained for the liver SWS with the three different network types.

Network	VOI (ml)	SWS (m/s)	Dice score	HD (voxel)	ICC	Pearson coefficient
Ground truth	541 ± 157	1.35 ± 0.13	–	–	–	–
Pretrained	537 ± 151 (n.s.)	1.35 ± 0.12 (n.s.)	0.94	7.87	0.99	0.99 (***)
2D U-Net	526 ± 146 (n.s.)	1.35 ± 0.12 (n.s.)	0.95	8.06	0.99	0.99 (***)
3D U-Net	533 ± 148 (n.s.)	1.35 ± 0.13 (n.s.)	0.94	7.68	0.99	0.99 (***)

*VOI*, volume of interest; *HD*, Hausdorff distance; *ICC*, intraclass correlation coefficient; *n.s.*, not statistically significant; *, *p* ≤ 0.05; **, *p* ≤ 0.01; ***, *p* ≤ 0.001.

### Spleen

**Fig. 8:**
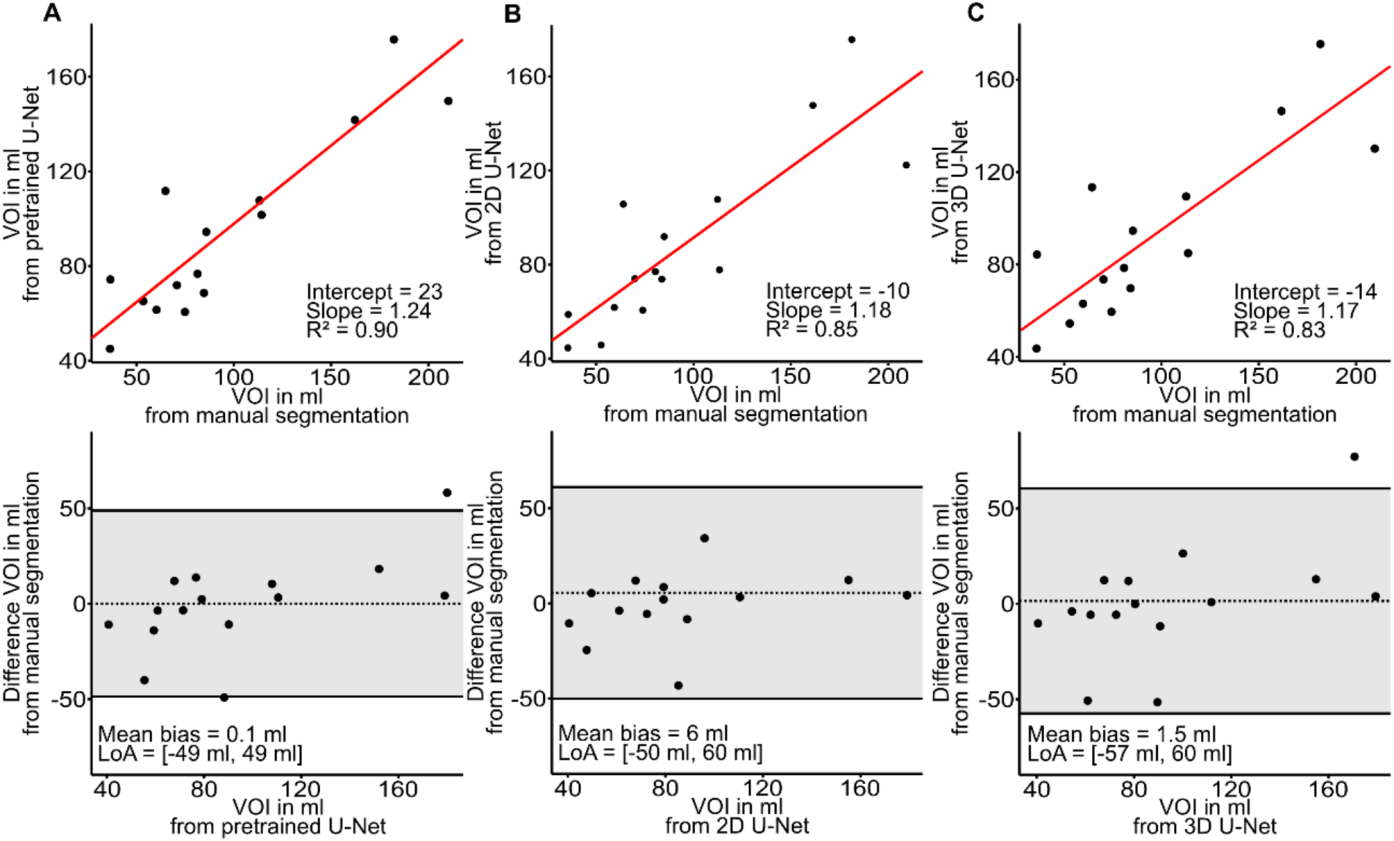
Spleen. Agreement between three different U-Nets and ground truth for VOI. Linear regression and Bland-Altman analysis for segmentation computed by pretrained network **(a)**, by 2D U-Net **(b)**, and by 3D U-Net **(c)**.

Mean spleen VOI size was 94 ± 50 ml, 94 ± 36 ml, 89 ± 36 ml, and 93 ± 36 ml for ground truth, pretrained, 2D U-Net, and 3D U-Net, respectively, without a statistically significant difference (*p* = 0.95). Figure 8 shows VOI values from manual segmentation compared with those obtained from U-Nets. As shown in Fig. 5, the mean spleen Dice score was 0.89, 0.90, 0.89, while mean spleen Hausdorff distance was 6.71 voxels, 6.16 voxels, and 6.25 voxels for pretrained, 2D U-Net, and 3D U-Net, respectively. Subgroup analysis based on field strength, breathing pattern, and frequency revealed no statistically significant differences in Dice scores. Mean spleen SWS was 1.94 ± 0.21 m/s, 1.92 ± 0.21 m/s, 1.93 ± 0.22 m/s, and 1.92 ± 0.22 m/s for ground truth, pretrained, 2D U-Net, and 3D U-Net, respectively, without a statistically significant difference (*p* = 0.99). Pairwise comparisons of *p*-values of post-hoc analysis are listed in Table 3. For the spleen, we observed similar uncertainty feature boundaries as for the liver. For incorrectly predicted areas on the left and right side, there was a coarser and more highlighted boundary. The pretrained model’s segmentation was challenging for the last slice, which is reflected in the homogenous uncertain area highlighted by the red arrow in Fig. 6.

**Fig. 9:**
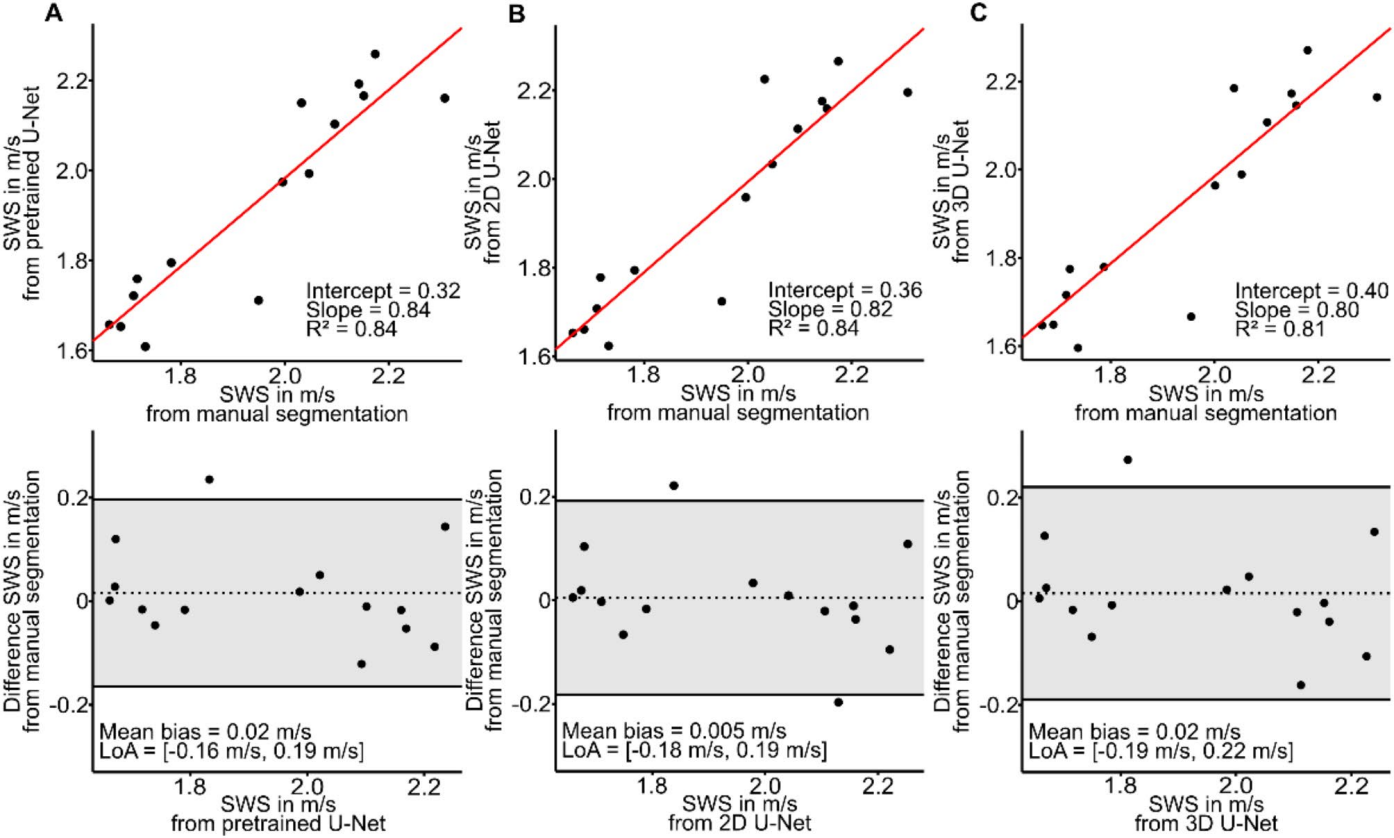
Spleen. Agreement between three different U-Nets and ground truth for SWS. Linear regression and Bland-Altman analysis for segmentation computed by pretrained network **(a)**, by 2D U-Net **(b)**, and by 3D U-Net **(c)**.

Figure 9 shows averaged SWS values obtained from manual segmentations compared with those obtained from U-Nets. Mean SWS values from manual segmentation correlated with the mean values from the pretrained network (*R* = 0.84, slope = 0.84, intercept = 0.32), 2D U-Net (*R* = 0.84, slope = 0.82, intercept = 0.36), and 3D U-Net (*R* = 0.81, slope = 0.80, intercept = 0.40). There was a bias of 0.02 m/s, 0.005 m/s, and 0.02 m/s for pretrained, 2D U-Net, and 3D U-Net, respectively. 95% LoA were (− 0.16) to 0.19 m/s, (− 0.18) to 0.19 m/s, and (− 0.19) to 0.22 m/s for pretrained, 2D U-Net, and 3D U-Net, respectively. The ICC was 0.92, 0.92, and 0.90 for pretrained, 2D U-Net, and 3D U-Net, respectively.

**Table 3:** Summary of parameters and post-hoc *p*-values (in parentheses, pairwise comparison with ground truth) obtained for the spleen SWS with the three different network types.

Network	VOI (ml)	SWS (m/s)	Dice score	HD (voxel)	ICC	Pearson coefficient
Ground truth	94 ± 50	1.94 ± 0.21	–	–	–	–
Pretrained	94 ± 36 (n.s.)	1.92 ± 0.21 (n.s.)	0.89	6.71	0.92	0.92 (***)
2D U-Net	89 ± 36 (n.s.)	1.93 ± 0.22 (n.s.)	0.90	6.16	0.92	0.92 (***)
3D U-Net	93 ± 36 (n.s.)	1.92 ± 0.22 (n.s.)	0.89	6.25	0.90	0.90 (***)

*VOI*, volume of interest; *HD*, Hausdorff distance; *ICC*, intraclass correlation coefficient; *n.s.*, not statistically significant; *, *p* ≤ 0.05; **, *p* ≤ 0.01; ***, *p* ≤ 0.001.

## Discussion

In this study, we have demonstrated the feasibility of U-Net-based automated segmentation of liver and spleen in MRE magnitude images. We have found an excellent agreement between manual and automated segmentations. For liver segmentation, best performance was found for the 2D U-Net, although Dice scores of the 3D U-Net and pretrained U-Net were comparably high. The 2D U-Net al.so performed best for spleen segmentation; again, although Dice scores of the 3D U-Net und pretrained U-Net were comparably high. The use of a pretrained U-Net showed no advantage for either liver or spleen segmentation. Subgroup analysis showed no statistically difference in dice scores, demonstrating the network’s capacity for generalization. Although segmentation performance was excellent for both liver and spleen, it was even slightly better for the liver. Spleen segmentation is often less accurate than that of the liver because the spleen is generally smaller than the liver and the former may exhibit similar signal intensity to that of the left kidney and pancreatic tail on the MRE magnitude images^36–39^. This can also be seen in the qualitative uncertainty results in Fig. 6, which align closely with the Dice Score segmentation results for each model type. This is highlighted by the prominent uncertainty near the lower boundary of the liver seen for the 3D U-Net and pretrained U-Net. Figure 5 shows this effect of the same model type in the Dice score accuracy compared to the 2D U-Net. Uncertainty was higher for the spleen than for the liver, which was associated with less accurate mean SWS values compared to those obtained with manual segmentation. Generally, low spatial resolution can increase uncertainty, especially near segmentation boundaries. However, the networks performed quite well despite the low spatial resolution. Since the model was trained on magnitude images, the volumes of interest (VOIs) can be evaluated in parallel within an MRE image postprocessing pipeline, for instance available as open access at https://bioqic-apps.charite.de.

Wang et al. investigated a 2D U-Net for liver segmentation using a total of 330 CT and MRI scans. They found an excellent segmentation accuracy similar to our results with Dice scores of 0.94 for CT, 0.95 for T1-weighted MRI, and 0.92 for T2*-weighted MRI^15^. Meddeb et al. trained a 3D U-Net on a CT dataset including 61 patients with conditions that directly or indirectly affect the spleen. Similarly, they found an excellent segmentation accuracy for the spleen with a Dice score of up to 0.941^40,41^. Aldoj et al. investigated automated quantification of MRE parameters in prostate zones using Dense U-Net segmentation in 40 patients with benign prostatic hyperplasia or prostate cancer^20^. They found the best segmentation performance for MRE magnitude images alone (Dice scores of 0.92 for whole prostate, 0.91 for central zone, and 0.77 for peripheral zone) compared to magnitude images combined with T2-weighted and diffusion-weighted images (Dice scores of 0.91 for whole prostate, 0.91 for central zone, and 0.63 for peripheral zone) or T2-weighted images alone (Dice scores of 0.92 for whole prostate, 0.91 for central zone, and 0.65 for peripheral zone). Dzyubak et al. investigated automated calculation of stiffness to stage hepatic fibrosis^13,14^. The performance of their automated algorithm for staging hepatic fibrosis was equivalent to an expert radiologist.

Our study has limitations. First, the number of participants was small. Second, only healthy participants were included. Therefore, we could not investigate how segmentation might be influenced by factors such as ascites, benign or malignant lesions, cholestasis, intraperitoneal adipose tissue deposits, fibrosis or massive hepatosplenomegaly. These factors could affect the contrast of MRE magnitude images and, in turn, impact segmentation accuracy. This limitation will be addressed in future studies, where the method will be tested on patient data to evaluate its performance in clinical populations. Thirdly, training was conducted on good quality MRE magnitude images. Performance may be less effective when applied to lower-quality images. To enhance segmentation accuracy and improve generalization, future studies will incorporate multicenter MRE data acquired with diverse imaging protocols. Finally, U-Nets have limitations as they are computationally intensive, necessitating substantial memory and processing power for initial training, which may preclude their use in settings with limited computational resources^42,43^. However once training is performed, subsequent segmentation of VOIs can be performed in seconds compared to 30 min to 1 h for manual segmentation. Furthermore, U-Nets can have problems with generalization, especially when faced with out-of-domain data or images with significant variability, which can degrade their performance^44,45^. Lastly, the model’s interpretability is limited despite its success. Using uncertainty estimation, we can judge prediction trustworthiness to some extent^32^. Nevertheless, completely understanding the internal workings and decision-making processes of U-Nets remains a challenge, which can be a significant drawback in medical image segmentation.

In conclusion, our results demonstrate an excellent performance for automated liver and spleen segmentation of MRE magnitude images and quantification of MRE parameters using U-Nets. The 2D U-Net performed best in both liver and spleen segmentation. Our results suggests that fully automated quantification of MRE parameters within anatomical regions is feasible by leveraging the previously unexploited anatomical information conveyed in MRE magnitude images.

## Data Availability

The datasets generated during and/or analysed during the current study are available from the corresponding author on reasonable request.
